# A rare case of leiomyosarcoma with a pleomorphic component of the sigmoid colon

**DOI:** 10.1093/jscr/rjae454

**Published:** 2024-07-11

**Authors:** Takahiko Omameuda, Masaru Koizumi, Yuzo Miyahara, Hiroyuki Kitabayashi, Mikio Shiozawa, Satoru Kondo, Shigeo Kawai, Masaaki Kodama

**Affiliations:** Department of Surgery, Tochigi Medical Center Shimotsuga, Tochigi 329-4498, Japan; Department of Surgery, Tochigi Medical Center Shimotsuga, Tochigi 329-4498, Japan; Department of Surgery, Tochigi Medical Center Shimotsuga, Tochigi 329-4498, Japan; Department of Surgery, Tochigi Medical Center Shimotsuga, Tochigi 329-4498, Japan; Department of Surgery, Tochigi Medical Center Shimotsuga, Tochigi 329-4498, Japan; Department of Surgery, Tochigi Medical Center Shimotsuga, Tochigi 329-4498, Japan; Department of Pathology, Tochigi Medical Center Shimotsuga, Tochigi 329-4498, Japan; Department of Surgery, Tochigi Medical Center Shimotsuga, Tochigi 329-4498, Japan

**Keywords:** leiomyosarcoma, sigmoid colon, pleomorphic component

## Abstract

A 66-year-old man presented to our institution with a positive fecal occult blood test and lower abdominal pain. Although a tumor was found in the sigmoid colon, biopsy and imaging studies failed to enable the diagnosis of the cancer, and the patient underwent surgery for treatment and diagnosis. The tumor had two distinct areas with differing features shown both histopathologically and on imaging; it was thus diagnosed as a leiomyosarcoma of the sigmoid colon with a pleomorphic component. Here, we describe a rare case of leiomyosarcoma of the sigmoid colon with a pleomorphic component. There are no reports of leiomyosarcoma with pleomorphic components arising in the colon in the literature; thus, the recurrence and metastatic characteristics are unknown. Therefore, accumulating cases in the literature may provide valuable insights into diagnosing and treating these rare tumors.

## Introduction

Primary leiomyosarcoma of the colon is a rare cancer arising from the muscularis mucosae or muscularis propria of the large bowel with highly aggressive behavior and poor prognosis [1]. Approximately 8% of soft tissue leiomyosarcomas exhibit a nonspecific, poorly differentiated, pleomorphic appearance in addition to typical areas; these tumors are called pleomorphic or dedifferentiated leiomyosarcomas. Specifically, the term ‘dedifferentiated’ has been used for pleomorphic leiomyosarcomas that lack immunohistochemical staining for myogenic markers in the pleomorphic areas [2].

In this case, we encountered a case of a sigmoid colon tumor that presented a challenging preoperative diagnosis but was resected and ultimately pathologically diagnosed as a rare leiomyosarcoma with a pleomorphic component.

## Case report

**Figure 1:**
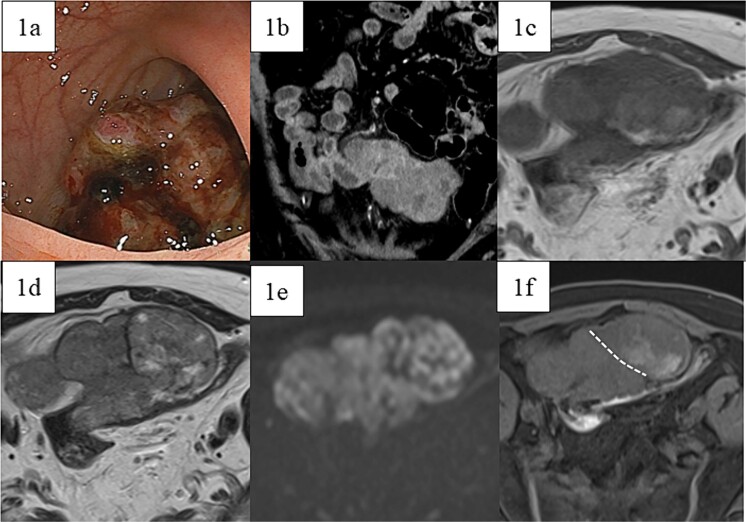
Preoperative images. (a) Lower gastrointestinal endoscopy revealed a type 1 tumor in the sigmoid colon. (b) Contrast-enhanced computed tomography showed a contrast-enhanced soft-tissue tumor measuring 80 mm × 95 mm × 45 mm (arrow), which was partially adherent to the small intestine. MRI showed a tumor that was heterogeneous in both (c) T1- and (d) T2-weighted images with predominantly low-intensity areas, with some high-intensity areas in T2. (e) Diffusion-weighted images showed high intensity throughout the entire tumor. Contrast-enhanced MRI (f) showed two areas with slightly different contrast effects. White dotted lines indicate boundaries of the areas with different contrast effects.

A 66-year-old male with a positive fecal occult blood test and lower abdominal pain underwent a lower gastrointestinal endoscopy, revealing a type 1 tumor in the sigmoid colon (Fig. 1a), though biopsies were inconclusive. Contrast-enhanced CT showed an 80 mm × 95 mm × 45 mm contrast-enhanced tumor adherent to the small intestine, with unclear origin (Fig. 1b). Magnetic resonance imaging (MRI) indicated a heterogeneous tumor with low-intensity areas on T1 and T2 and high intensity on diffusion-weighted images (Fig. 1c–e). Contrast-enhanced MRI revealed two areas with different contrast effects (Fig. 1f). Differential diagnoses included adenocarcinoma, GIST, sarcomas, and lymphomas, leading to resection.

**Figure 2:**
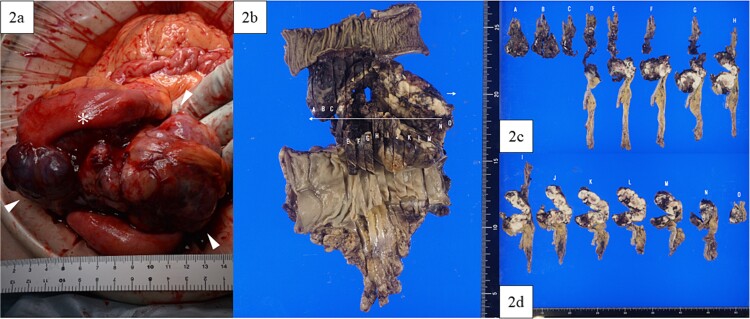
Intraoperative findings and macroscopic study of the surgical specimen. (a) Intraoperative findings showed a tumor (arrowhead) arising from the sigmoid colon and adherent to the small intestine (asterisk), with a white and solid area and a dark red and friable area. (b) Macroscopic study of the surgical specimen after formalin fixation. The high anterior resected specimen with partial resection of the small intestine predominantly had white and solid components, with dark red and fragile components on the small intestinal side and in the lumen of the sigmoid colon. (c, d) All resection margins, including proximal, distal, and circumferential margins, were negative.

**Figure 3:**
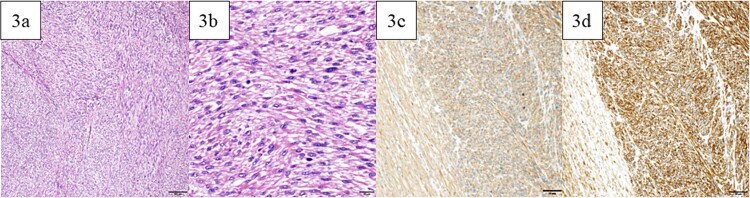
Pathological findings. (a) In the white and solid component, proliferating spindle-shaped cells with eosinophilic cytoplasm in intricate bundles were noted. (b) Uniform atypical spindle-shaped cells proliferated, and nuclear fission was observed in scattered images. The tumor cells were positive for αSMA (c) and desmin (d). Bars indicate 100 μm in Fig. 5a, 20 μm in Fig. 5b, and 50 μm in Fig. 5c and d.

**Figure 4:**
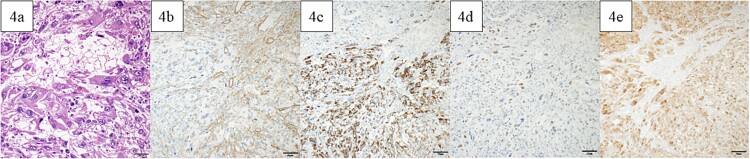
Pathological findings. In the dark red and fragile component, a high degree of atypical and pleomorphic tumor cell growth was observed (a). The tumor cells were positive for αSMA (b), desmin (c), MDM2 (d), and CDK4 (e). Bars indicate 50 μm in Fig. 6a–e.

A lower midline incision revealed a sigmoid colon tumor adherent to the small intestine (Fig. 2a). The surgery involved ligation of the inferior mesenteric artery distal to the left colic artery, manipulation of the sigmoid colon mesentery, and en bloc resection of the small intestine. The tumor had a white solid area and a dark red friable area, as seen on an MRI. Partial resection of the small intestine and high anterior resection were performed. The specimen had both white solid and dark red fragile components (Fig. 2b–d). Histopathology of the white areas showed spindle cells with eosinophilic cytoplasm and intersecting fascicles (Fig. 3a and b). The tumor cells were positive for αSMA and desmin (Fig. 3c and d) but negative for CDK4, MDM2, and DOG1. The dark red areas had markedly atypical cells resembling high-grade pleomorphic sarcoma (Fig. 4a) and were positive for αSMA, desmin, MDM2, and CDK4 (Fig. 4b–e). The tumor lacked well-differentiated liposarcoma but had a low-grade leiomyosarcoma component, diagnosed as leiomyosarcoma with a pleomorphic component according to WHO Classification (2020).

**Figure 5:**
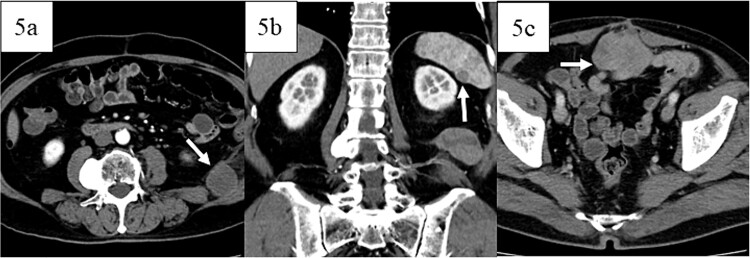
Images of contrast-enhanced computed tomography, contrast-enhanced computed tomography scan revealed recurrence in the retroperitoneum 12 months postoperatively (white arrow in 5a) and metastases in the spleen 16 months postoperatively (white arrow in 5b), and peritoneal dissemination (white arrow in 5c) 27 months postoperatively.

Postoperatively, the patient developed paralytic ileus, resolving with conservative treatment, and was discharged 21 days after surgery. Adjuvant therapy was not administered because the patient had a complete resection with clear margins. Twelve months later, retroperitoneal recurrence was noted (Fig. 5a), and doxorubicin was started. Sixteen months post-surgery, splenic metastasis was detected (Fig. 5b). After five cycles of doxorubicin, the patient had progressive disease but refused invasive treatment and received supportive care. Peritoneal dissemination was identified 27 months post-surgery on CT (Fig. 5c). The patient died 3 years and 2 months post-operation.

## Discussion

Sarcomas are malignant tumors that arise in the connective tissues, account for <1% of all adult cancers, and arise in the soft tissues in at least 70% of cases [3]. The most common sites of digestive leiomyosarcomas are the small intestine (45%) and the colon (38%) [4]; these tumors are most often diagnosed in middle-aged patients (50–60 years old), with a slight male preponderance [4].

The presence of activating KIT mutations in 94% of GISTs was first reported by Hirota *et al.* [5] in 1998. The KIT gene, a tyrosine kinase receptor proto-oncogene, increases cell proliferation, and its mutation gene leads to cellular atypia and neoplasia. Later studies confirmed that 95% of GISTs express CD34, CD137, and DOG1 [6]. In contrast, leiomyosarcoma is negative for KIT mutations and mostly positive for desmin, SMA, h-caldesmon, and vimentin [7]. The distinction between leiomyosarcoma and GISTs is of paramount importance, as these types have very similar clinical presentations but require radically different treatment approaches. The cellular origin of GISTs—i.e. the interstitial cells of Cajal—was first described in the mid-1990s. However, leiomyosarcoma originates from the muscle fibers of the muscularis mucosa and muscularis propria [8].

MRI typically shows a voluminous mass that is hypointense on T1 and hyperintense on T2, with central T2 hyperintense areas of necrosis and heterogeneous enhancement [9]. A definitive diagnosis is made only after histological examination of the biopsy or resected specimens.

As the standard of care for local soft tissue and visceral sarcomas [10], surgery is performed in all cases; however, no standard therapeutic strategy for gastrointestinal leiomyosarcoma has yet been established. While lymph node metastasis of gastrointestinal leiomyosarcomas is rather uncommon, lymph node dissection is advisable if it is not excessively invasive, as lymph node metastasis has been reported even in small and poorly proliferating tumors (one such tumor was 1 cm in diameter and had 18 mitoses/50 high-power fields) [11]. Standard chemotherapies for advanced soft tissue and visceral sarcomas include first-line anthracyclines with doxorubicin plus dacarbazine as an alternative [10]. In this case, doxorubicin monotherapy was selected after consideration of the side effects. Chemotherapy has a limited role in the treatment of leiomyosarcoma. The prognostic factors of leiomyosarcoma are unclear because leiomyosarcoma is rare, but a tumor diameter ≥5 cm has been reported to have a poor prognosis [11].

This case initially suggested dedifferentiated liposarcoma with smooth muscle differentiation due to strong MDM2 and CDK4 positivity. However, the absence of a highly differentiated liposarcoma component or fatty tumor suggested leiomyosarcoma instead. Contrast-enhanced MRI revealed distinct tumor contrast, indicating two diverse components. Macroscopically and pathologically, these components differed, leading to the diagnosis of leiomyosarcoma with a pleomorphic component in the sigmoid colon. No previous cases of colon leiomyosarcoma with a pleomorphic component have been reported. Monitoring which component recurs or metastasizes may be valuable. The patient experienced recurrence in the retroperitoneum at 12 months, splenic metastasis at 16 months, and peritoneal dissemination at 27 months postoperatively. The retroperitoneal and splenic lesions were homogeneous, while peritoneal dissemination had an uneven contrast on CT. Though neither lesion was biopsied, they may have originated from different components. More cases could clarify the relationship between recurrence/metastasis patterns and imaging/pathological findings. This report highlights a rare leiomyosarcoma with a pleomorphic component in the sigmoid colon, warranting further case studies to understand the recurrence and metastasis patterns of leiomyosarcoma of the colon.

## Data Availability

Not applicable.
